# Game Indicators Determining Sports Performance in the NBA

**DOI:** 10.2478/hukin-2013-0035

**Published:** 2013-07-05

**Authors:** Kazimierz Mikołajec, Adam Maszczyk, Tomasz Zając

**Affiliations:** 1 Department of TeamSports, Academy of Physical Education in Katowice, Poland.; 2 Department of Sports Theory, Academy of Physical Education in Katowice, Poland.; 3 Human Performance Laboratory, Academy of Physical Education in Katowice, Poland.

**Keywords:** basketball, NBA, performance variables, regression model, optimization

## Abstract

The main goal of the present study was to identify basketball game performance indicators which best determine sports level in the National Basketball Association (NBA) league. The research material consisted of all NBA game statistics at the turn of eight seasons (2003–11) and included 52 performance variables. Through detailed analysis the variables with high influence on game effectiveness were selected for final procedures. It has been proven that a limited number of factors, mostly offensive, determines sports performance in the NBA. The most critical indicators are: Win%, Offensive EFF, 3rd Quarter PPG, Win% CG, Avg Fauls and Avg Steals. In practical applications these results connected with top teams and elite players may help coaches to design better training programs.

## Introduction

Statistics in sports have been an important tool for coaches to evaluate the team and player sports performance ( Hughes and Franks, 2004 ; Ortega et al., 2009 ; Leite et al., 2009 ; Oliver, 2004). Throughout the years of competitive basketball, numerous methods of game registration and analysis have been created, with the objective to precisely and objectively evaluate particular players and the whole team. These methods evolved from simple stat sheets, filled out by hand during the game by assistant coaches to fully computerized procedures that automatically register all of the significant variables of the game and calculate the necessary indices ( Lorenzo et al., 2010 ; Oliver, 2004). Currently, basketball is one of the most analyzed sport disciplines. The analyses of the statistical reports allow coaches to evaluate the technical and tactical efficiency of players and teams, and to compare them during single game performance, as well as during the whole season. They also help players to develop basketball skills based on recorded factors ( Gomez et al., 2009 , 2010 ; Ibanez et al., 2008 ; Sampaio and Janeira, 2003 ; Oliver, 2004). Currently the NBA (National Basketball Association) registers games and performs statistical analysis of them including the smallest details (Oliver, 2004).

The obtained data consist of information of particular players and teams. The winner of the NBA competition is unofficially classified as a world champion. For that reason recorded statistics have been so valuable for further analysis. Team statistics are related to the level of tactical preparation and game strategy

The investigation in this area has been connected with several issues: game efficiency depends on gender (male, female), age (kadet, senior), and sport performance (national leagues, Euroleague, NCAA, World Chapionship, Olimpic Games), comparison of winning and losing teams ( Trninić et al., 2002 ; Kozar et al., 1994 ; Lorenzo et al., 2010 ) and different parts of the season – regular, play-offs (Sampio and Janeira, 2002; Oliver, 2004), as well as player’s court position. Some research has also analyzed game performance as a function of a competition phase (Oliver, 2004; Gomez et al., 2008 ) or tactical strategies ( Gomez et al., 2008 ; Oliver, 2004). Several studies have been connected with the difference in the final score (close, balanced and unbalanced games).

Unfortunately, the number of considered games and variables have been restricted. Also a limited number of studies have been conducted on top-level competition. Therefore, the aim of the present study was to identify basketball game performance indicators which best determine sports level in the NBA.

## Material and Methods

### Sample and variables

The data were obtained from the National Basketball Association (NBA) (official statistics gathered live) for the seasons 2003–11. All data were collected from official boxscores of NBA and included 52 variables that characterized offensive and defensive efectiveness of 30 teams. Through a detailed analysis of these variables, we excluded those that did not have a significant effect on game performance and selected those that had a high impact for final analysis. The dependent variable was the result achieved by a particular team in a certain season (Y). Eight NBA seasons (2003–11) were taken into consideration in the present study.

The empirical, prognostic and exploratory studies as well as model econometrics, factor analysis and cluster analysis as statistical methods were used in this research ( Maestas and Preuhs, 2000 ). The scheme of the study included the following structure of variables: RXn ^n^ Y ^n^ (one multivalent dependent variable Y ^n^ and n multivalent independent variables Xn ^n^ respecting the rule of purposeful selection ). Descriptive statistics were used to compare the correlations of variables and to determine the most important ones for data prediction and mathematical modeling.

The distribution of all variables for each NBA team was verified. The analysis of calculated coefficients of variance (V) revealed the largest diversity in the following variables: number of ball possessions, average score per game, number of scored points in the first quarter, average number of 3 point shots made, average biggest point advantage, total number of shots made, 3 point shots made, average number of fouls. Very similar diversity was observed in all NBA teams. Due to the skewness index all variables were characterized by normal distribution with moderate right or left asymmetry. According to scientific procedures ( Mc Cullough and Wilson, 2005 ; Keele and Kelly, 2006 ) the agglomeration Ward method was used in order to group the subjects. All teams were divided into five groups: 1/ Denver, Golden State, Phoenix, 2/ Dallas, LA Lakers, Sacramento, 3/ Boston, Cleveland, Houston, Indiana, Miami, Milwaukee, New York, Okla City, Orlando, San Antonio, Toronto, Washington, 4/ Detroit, New Jersey, New Orleans, Portland, 5/ Atlanta, Charlotte, Chicago, LA Clippers, Memphis, Minnesota, Philadelphia, Utah (the order is random). Then the relationship between all variables was defined by means of the Pearson’s correlation coefficient (r) taking into consideration the evaluated years. Due to a large number of variables with a statistically significant value of the Pearson’s coefficient (48), their number was reduced using factors analysis and appointed the vectors R1 and R0. The correlation coefficients for 29 explanatory variables were calculated according to the following formula:

$$r_{\mathrm{ij}}=\frac{\sum_{t=1}^{n}\left(x_{\mathrm{ti}}-{\bar{x}}_{i}\right)\left(x_{\mathrm{tj}}-{\bar{x}}_{j}\right)}{\sqrt{\sum_{t=1}^{n}{\left(x_{\mathrm{ti}}-{\bar{x}}_{i}\right)}^{2}\sum_{t=1}^{n}{\left(x_{\mathrm{tj}}-{\bar{x}}_{j}\right)}^{2}}}\;\;\;\;\;\;\;\;\;\;\;\;\;\;\;\;\;\;\;\;\;\left(\text{i},\text{j}=1,2,\ldots ,\text{m}\right)$$

Thus, the relationship between analysed variables and matrix of correlation coefficients between explanatory variables and the vector of the explanatory variable Y with potential explanatory variables were determined as presented below:

$$r_{i}=\frac{\sum_{t=1}^{n}\left(y_{t}-\bar{y}\right)\left(x_{\mathrm{ti}}-{\bar{x}}_{i}\right)}{\sqrt{\sum_{t=1}^{n}{\left(y_{t}-\bar{y}\right)}^{2}\sum_{t=1}^{n}{\left(x_{\mathrm{ti}}-{\bar{x}}_{i}\right)}^{2}}}\;\;\;\;\;\;\;\;\;\;\;\;\;\;\;\;\left(\text{i}=1,2,\ldots ,\text{m}\right)$$

As a result, the vector R0 was determined. Then the method of correlation coefficients for determination of correlation matrix, correlation vectors and optimal predictants selection was used. The critical value of correlation coefficient was calculated by means of the following formula:

$$r^{*}=\sqrt{\frac{{\left(t^{*}\right)}^{2}}{{\left(t^{*}\right)}^{2}+n-2}}$$

For the level of significance α=0,1 and n=2 degrees of freedom, 30−2=28 the value of statistic and critical value was t=1,70, r* = 0.30, respectively. All variables with diagnosed inequality |r _j_ |≤ r*, were eliminated as not significantly correlated with the dependent variable Y. The obtained result was verified by factor analysis. Thus, the most correlated 29 variables were calculated. Using procedures described above, the optimal combination of variables was determined ( Table 1 ).

The obtained model explained 99% of variability which proves its adjusting to the input data. The matrix set included 29 variables divided into five groups of factors. Thus, the optimal combination of variables in order to create the regression models was selected.

### Statistical Analysis

The most common and comprehensively verified statistical methods were used to optimize the conclusions of the analyses which were carried out in this study. The intercorrelations between analysed variables were calculated by the Pearson’s coefficient ( Ferguson and Takane, 1997 ; Maestas and Preuhs, 2000 ; Green, 2003 ; Keele and Kelly, 2006 ). According to sport results, the regession analysis was used ( Jaccard, 1990 ; Ginevan and Splitstone, 2004 ). Identification of the optimal combination of explanatory variables were done by the correlation matrix of variables, Pearson’s coefficient and factor analysis ( Green, 2003 ; McCullough and Wilson, 2005 ; Keele and Kelly, 2006 ). The impact of variables on the value of Y (explanatory variable) was analysed by multivariate function of regression with parameters calculated by the data characterizing the structure of following function:

$$Y_{t}=f\left[X_{1,t-1},X_{2,t-1},\ldots \ldots ,X_{n,t-1}\right]+\xi_{t}$$

After the simpification, the biometric model took the following structure:

$$Y=\sum_{i=1}^{k}\alpha_{j}x_{j}+\xi Y$$

The above presented statstical analysis was completed by Statistica PL including module Neural Networks (StatSoft Poland) and Excel Microsoft Office 2010 software (Microsof Poland).

## Results

The optimal combination for all NBA teams included 29 variables. In the next stage the regression analysis of results in the league as the dependent variable was conducted for chosen independent variables. The results are presented in Table 2 .

Due to this procedure the following structural parameters in the form of the equation of regression were revealed:

$$\begin{array}{l} (Y)=22,868+59,08\;\mathrm{Win}\;\%+0,18\;\mathrm{Avg}\;\mathrm{Fauls}+21,33\;\mathrm{Offensive}\;\mathrm{EFF}+2,46\;\mathrm{Win}\%\;\mathrm{CG}+3\mathrm{rd}\;\mathrm{Qrt}\;\mathrm{PPG}+0,28\;\mathrm{Avg}\;\mathrm{Steals} \end{array}$$

The statistically significant predictors of team‘s rank position are variables in weight order by value of Beta index: Win % (percent of wins during the whole season), Offensive EFF (offensive efficiency), 3rd Quarter PPG (average number of points in the 3rd quarter), Win % CG (percent of wins in the close games), Avg Fauls (average number of fauls) and Avg Steals (average number of steals). The variables explained 86% of variance for Y (dependent variable) and the multiple correlation between exogenous variables and endogenous one was equal 0,93.

Additionally, the verification of the model indicates that an increase in any parameter would improve ranking. For example if the number of wins during the NBA season changes positively by 1% then the team would receive 50 ranking points more.

## Discussion

The literature review connected with sports performance in basketball indicates on a limited number of considered variables and games. Furthermore, a restricted number of studies have been conducted on top-level competition. Therefore, the aim of the present study was to identify basketball game performance indicators which best determine sports level in the NBA and wether they remain stable or change from season to season.

The study included 52 performance variables, both offensive and defensive. Through a detailed analysis the variables that did not have a significant impact on game performance were excluded. After completing a series of mathematical calculations the most important game indicators were selected for further analysis. It seems that each new season in the NBA is different from the previous one taking into account the factors influencing the game results. Meanwhile, it has been proven that championships in the NBA have been determined for the past few years by the same factors. These factors change only slightly in strength and their relationships. The most critical indicators which determinate the success at the end of the eight considered NBA seasons were: Win %, Offensive EFF, 3rd Quarter PPG, Win % CG, Avg Fauls and Avg Steals. The combination of variables presented above differs from the results obtained by other authors. Csataljay et al. (2009) revealed 6 factors having the highest impact on the final score in basketball game: 3 PTA (3 points attempts), FG % (field goals efficiency), FTM (free throws made), FT% (free throws efficiency), D-REB (number of defensive rebounds), TOR (number of turnovers). The research was conducted based on Euroleague statistics.

The correlation between the number of assists and win/defeat ratio was the aim of the study by Melnik (2001). The sample included NBA games from 5 seasons (1993–98). By means of the Spearman’s coefficient of rank correlation a significant relationship between these variables was shown (from 0,42 to 0,71). The best results in the NBA league were observed in teams playing best offense and defense. Ibáñez et al. (2003) analyzed 870 games of the Spanish league - LEB 1 (6 seasons). Obtained data showed that the result was affected by three main factors: assists, steals and blocks which explained 82,4% of the dependent variable. The number of assists was the most important variable determining success.

The results of many studies ( Akers et al., 1991 ; Ittenbach and Esters, 1995 ; Karipidis et al., 2001 ) confirmed that winning teams achieved better shooting efficiency and a higher number of defensive rebounds. Besides, Kozar et al. (1994) revealed that according to close games, the number of fouls and free throws effectiveness was most significant. The data presented by some authors indicate small influence of offensive rebounds, steals, turnovers and assists on basketball performance. It suggests that winning teams base their game on correct decisions, shooting efficiency and better strategies and tactics used in competition ( Trninić et al., 2002 ). Sampaio and Janeira (2003) indicated the importance of defensive rebounds which increased the number of ball possessions, what resulted in an opportunity for higher percentage of offensive actions.

Oliver (2004) selected 4 factors most affecting sport results in basketball: shooting efficiency, number of turnovers, offensive rebounds and free throws made. It was stated that winning teams achieved a high level in three out of the four listed above variables. The same author suggested that effective offensive play led to success in the NBA (Oliver, 2004). The best teams in this area won 79% of games. The high number of missed shots created possible fast break opportunities for opponents. Additionally, too many turnovers decreased the number of possessions and had an important influence on field goal attempts. Another aspect was an increased number of offensive rebounds which allowed to score more points. Due to this factor best teams can significantly improve their offensive efficiency. A large number of free throw attempts allowed to score more points with high-effective shots. It also created foul troubles and mandatory substitutes for the opponent (Oliver, 2004; Sampaio et al., 2008 ).

The development of set plays ( Gomez et al., 2008 ; Trninić et al., 2002 ), number of assists (Hoofler and Payne, 1997), higher level of physical fitness ( Royal et al., 2006 ), number of defensive rebounds as a factor limiting the opposition’s chances to score ( Gomez et al., 2008 ; Hoofler and Payne, 1997) are important elements of game statistics that mostly influenced game results. Gomez et al. (2008) also stressed the importance of 3 points shots that can make a major difference between winning and losing teams. However, obtained results pointed out that in balanced games the teams had to be more effective in short distance field goals to win the match. The conducted research also revealed a small importance of assists in playoff games. This can be explained by a less frequent teamwork and more individual plays selected by the best players in decisive games.

It can be concluded that the final game result is affected by many variables, which may not always be important during particular matches, but constant effort to maintain them at the highest level gives an advantage over less organized teams.

The main factors which influence sports results in the NBA indicated in the present study are much more connected with offense than defense. It suggests that this area of basketball game is more important. Most conclusions defined by other authors and presented above are similar and confirm results obtined in this research. However, the importance of individual factors can be expressed in a different way. In practical applications, these results connected with top teams and elite players may help coaches to design better training programs.

## Figures and Tables

**Table 1:** *
The results of factor analysis for 30 NBA teams
*

Variables	Factor	Factor	Factor	Factor	Factor
	1	2	3	4	5
Win% Season	0,087	0,936	0,077	0,191	−0,066
Win% Home	0,123	0,894	0,094	0,097	−0,107
Win% Away	0,038	0,862	0,050	0,266	−0,014
Win% CG (close games)	−0,079	0,710	0,057	0,005	−0,064
Possesion P.G	0,864	−0,328	0,096	0,143	−0,202
Possesion P.G Home	0,842	−0,301	0,124	0,135	−0,215
Possesion P.G Away	0,843	−0,337	0,060	0,142	−0,182
PPG	0,919	0,234	0,080	0,267	0,065
PPG Home	0,892	0,242	0,092	0,228	0,031
PPG Away	0,883	0,209	0,061	0,292	0,100
1st Qrt PPG	0,831	0,263	0,031	0,232	0,022
2nd Qrt PPG	0,843	0,164	0,009	0,237	0,056
3rd Qrt PPG	0,788	0,278	0,077	0,229	0,013
4th Qrt PPG	0,728	0,115	0,175	0,241	0,124
2 Pts Avg	0,635	0,081	−0,095	−0,744	0,042
3 Pts Avg	0,336	0,150	−0,158	0,907	0,065
Avg Biggest Lead	0,179	0,739	0,065	0,206	−0,066
FGM	0,931	0,210	−0,229	0,023	0,098
FGA	0,732	−0,243	−0,429	0,058	−0,226
3 Pts Made	0,336	0,152	−0,158	0,906	0,064
3 Pts Att.	0,310	0,081	−0,155	0,915	0,017
FTM	0,295	0,076	0,800	−0,114	−0,072
FTA	0,216	0,035	0,906	−0,139	−0,075
3 Pts %	0,197	0,126	−0,092	0,948	0,059
2 Pts %	−0,197	−0,126	0,092	−0,948	−0,059
Free Throw %	−0,039	0,106	0,943	−0,139	0,011
FTA per Offensive Play	−0,019	0,154	0,918	−0,138	0,024
Stls (steals)	0,210	0,027	0,124	−0,109	−0,852
Stls per Defensive Play	−0,015	0,131	0,112	−0,147	−0,820

**Table 2:** *
Summary of regression for dependent variable – NBA rank for 30 teams
*

N=240	R= ,982 R^2= ,964			R2= ,963		
	F(6,233)=1059,5 p<0,0000 Std. dev. error of estimat.: 1,6518					
	b*	St. error	b	St. error	t	p
Intercept			22,865	5,129	4,457	0,001
Win %	1,032	0,020	59,081	1,194	49,456	0,001
Avg Fauls	0,034	0,013	0,188	0,073	2,562	0,011
Offensive EFF	0,084	0,024	21,333	6,262	3,406	0,003
Win % CG	0,035	0,0159	2,464	1,092	2,255	0,025
3rd Qrt PPG	0,045	0,019	0,303	0,131	2,302	0,022
Avg Steals	0,027	0,013	0,284	0,137	2,066	0,031
